# The Utility of Umbilical Cord Pulse Oximetry—A Translational Study with Four Minutes of Deferred Cord Clamping Using an Asphyxiated Preterm Ovine Model

**DOI:** 10.3390/children12091205

**Published:** 2025-09-10

**Authors:** Justin Helman, Mausma Bawa, Sylvia Gugino, Nicole Bradley, Lori Nielsen, Arun Prasath, Clariss Blanco, Mary Divya Kasu, Hamza Abbasi, Munmun Rawat, Praveen Chandrasekharan

**Affiliations:** 1 Division of Neonatology, Department of Pediatrics, University at Buffalo, Buffalo, NY 14203, USA; jhelman@buffalo.edu (J.H.); mausmaba@buffalo.edu (M.B.); sfgugino@buffalo.edu (S.G.); nkbradle@buffalo.edu (N.B.); lnielsen@buffalo.edu (L.N.); hamzaabb@buffalo.edu (H.A.); munmunra@buffalo.edu (M.R.); 2 Department of Pediatrics, Boston Children’s Hospital, Harvard Medical School, Boston, MA 02115, USA; 3 Division of Neonatology, Department of Pediatrics, UT Southwestern, Dallas, TX 75390, USA; arun.prasath@utsouthwestern.edu; 4 Division of Neonatology, Department of Pediatrics, Harlem Hospital, New York, NY 10037, USA; clarissblanc@gmail.com; 5 Division of Neonatology, Department of Pediatrics, University of Maryland, College Park, MD 20742, USA; mkasu@som.umaryland.edu

**Keywords:** preterm pulse oximetry, deferred cord clamping, umbilical pulse oximetry

## Abstract

**Highlights:**

**What are the main findings?**

- Umbilical pulse oximetry is a novel method to measure umbilical cord saturations in newborns.
- Umbilical saturations and umbilical heart rate through pulse oximetry, although postductal, had a significant correlation with arterial saturations and invasive carotid heart rate measurements.

**What is the implication of the main finding?**

- Umbilical cord pulse oximetry could serve as an alternative to measure oxygen saturation while undergoing a longer duration of deferred cord clamping.
- Umbilical cord pulse oximetry may help decide the duration of deferred cord clamping in the future.

**Abstract:**

Background: Expert guidelines recommend using pulse oximetry (PO) in the delivery room to monitor oxygen saturation (SpO_2_) and heart rate (HR). Umbilical cord pulse oximetry (UCP) is a novel concept that, despite being postductal, could provide accurate measurements of SpO_2_ and HR, as it overcomes barriers associated with skin pigmentation. Methods: This pilot study used NONIN pulse oximetry on an intact umbilical cord that underwent deferred cord clamping (DCC) to evaluate umbilical cord SpO_2_ in a preterm asphyxiated ovine model (N of 5) with an HR of <100 bpm. The UCP HR served as a surrogate marker for umbilical vessel flow. A receiver operator characteristic (ROC) curve was used to evaluate UCP parameters with arterial saturations (SaO_2_) and carotid HR between 2 and 10 min. Results: Following asphyxia, five preterm lambs underwent DCC for 4 min. A significant relationship was noted between SaO_2_ and umbilical SpO_2_ (area under the curve (AUC) of 0.907, CI 0.857–0.968, *p* < 0.0001) along with carotid and umbilical HR (AUC) of 0.842 (CI 0.663–0.902, *p* < 0.0001). Conclusions: In a translational preterm model, UCP accurately predicted preductal SaO_2_ and carotid HR (a surrogate for umbilical flow). Using UCP in the delivery room will help guide supplemental oxygen and determine the optimal duration of clamping the umbilical cord. These proof-of-concept studies/pilot findings require validation with larger animal cohorts and newborn infants.

## 1. Introduction

Premature neonates with immature lungs and surfactant deficiency often require supplemental oxygen (O_2_) that is titrated based on preductal saturations (pre SpO_2_), as recommended by the International Liaison Committee on Resuscitation (ILCOR) [1,2,3]. Pulse oximetry is a non-invasive and easy method to measure the saturation of O_2_ in tissues. A pulse oximeter uses a light source and sensor to measure the absorption of red light passing through capillaries in the skin to measure the O_2_ carried in the blood [4]. It estimates the percentage of hemoglobin carrying O_2_, and the monitor displays the oxygen saturation, which ranges from 0% to 100%, whose accuracy differs as lower values are observed [5]. Food & Drug Administration (FDA) and other studies have warned about the variability in pulse oximetry readings based on skin color, perfusion, temperature, etc. [6]. Dark-skinned infants are at high risk for hypoxemia due to consistent errors in SpO_2_ from pulse oximetry due to color [7], and the COVID-19 pandemic brought new attention to the issue of racial bias in pulse oximeters in adults [8]. Skin color variability and acrocyanosis in neonates can affect the pre SpO_2_. With good technique, a pulse oximeter will accurately display the heart rate and oxygen saturation within approximately 1 to 2 min of birth. In a clinical scenario involving a preterm infant with dark skin, poor skin development, and poor peripheral perfusion with acrocyanosis, umbilical pulse oximetry could be a useful tool. Although post-ductal, we introduce a concept called umbilical cord pulse oximetry that could offer a new approach to monitor SpO_2_ and HR without the discrepancies caused by skin involvement. It may also facilitate decisions about deferring cord clamping, thus improving neonatal outcomes.

Our objective was to assess the accuracy of umbilical cord pulse oximetry on (a) umbilical cord saturation (U SpO_2_) and (b) umbilical cord heart rate (U HR), which is also a surrogate for umbilical blood flow, as pulse oximetry detects pulsatile flow, in a preterm asphyxiated ovine model of deferred cord clamping for 4 min.

## 2. Materials and Methods

### 2.1. Preterm Ovine Model

Time-dated preterm pregnant ewes at 125–128 d (Polypay breed) gestation were studied as per ARRIVE guidelines [9] after approval by the Institutional Animal Care and Use Committee of the University at Buffalo. The preterm fetal lambs were instrumented while in placental circulation and with the ewes under general anesthesia. A jugular venous line was placed for access and blood draw. The right carotid artery was catheterized for pressure monitoring and arterial blood gas draws. Blood flow transducers were placed to monitor the left common carotid blood flow, left pulmonary artery blood flow, umbilical artery (UA), and vein (UV). Pulse oximetry saturation probes were placed around the right forelimb and tongue (pre SpO_2_), tail (post SpO_2_), and umbilical cord (U SpO_2_). The U SpO_2_ sensor was placed on the cord stump as close to the abdomen as possible. Removing excess blood from the delivery, on the cord before placement of probes, while appropriate, was not required for measures to be obtained. SaO_2_ from arterial blood gas (Radiometer ABL 825, Brea, CA, USA) was considered the gold standard for assessing U SpO_2_. We used the Nonin X-100 Sensmart with four sensors for our study. The heart rates (HRs) were collected from the carotid artery (gold standard).

### 2.2. Pulse Oximetry

Pulse oximetry saturation measurements were obtained from the preductal (right forelimb/tongue, whichever had a better signal), postductal (tail), and umbilical cord SpO_2_. Nonin SenSmart Model X-100 (Plymouth, MN, USA) uses PureSAT^®^ technology, which employs advanced pulse-by-pulse measurement. Pulse oximeters (like the Nonin X-100 SenSmart) use light absorption to estimate oxygen saturation: Two wavelengths of light (red and infrared) pass through tissue, and oxygenated hemoglobin (HbO_2_) absorbs more infrared light and lets more red light through. Deoxygenated hemoglobin (Hb) absorbs more red light and lets more infrared light through. A photodetector on the opposite side measures how much light gets through at each wavelength. The device calculates the ratio of red to infrared absorption and applies algorithms (like Nonin’s PureSAT^®^) to estimate arterial oxygen saturation (SpO_2_). The blood volume in tissue changes with each heartbeat. By detecting the “pulsatile” signal (arterial blood) and filtering out constant signals (skin, bone, venous blood), the oximeter specifically measures arterial oxygen saturation rather than all hemoglobin in the tissue. Using the above, SpO_2_ (%) is estimated as the percentage of hemoglobin molecules bound with oxygen in arterial blood, and pulse rate (BPM) → beats per minute, is detected from the same pulsatile signal. The probes were secured by being placed in a circular fashion and closed with Velcro around the umbilical cord, and a Posey pulse oximetry probe wrap was placed. Careful placement around the umbilical cord did not impact the umbilical arterial or umbilical venous flow. Blanchet et al. reported the variability between different pulse oximetry instruments [10]. Nonin Sen Smart™ Model X-100 has 4 pulse oximetry probes that can measure saturation and heart rate simultaneously, thus avoiding multiple saturation measuring instruments during ventilation while on the umbilical cord. They are used in some intensive care units that also use regional saturations around the world.

### 2.3. Protocol

Asphyxia was induced in preterm lambs via cord occlusion until the right carotid HR dropped below 90 beats per minute (bpm). Positive pressure ventilation (PPV) was initiated using an endotracheal tube (ETT) while the cord remained intact, and umbilical cord clamping occurred at 4 min. The measurements were part of another randomized study. As mentioned previously, we chose a particular arm of the study after randomization to stay consistent. In regard to the number of lambs, Law et al. & Allgoewer et al. have used 2 methods to come up with an N of 4–6 for pilot large animal studies, especially when continuous variables are used for analysis [11,12]. Our study is a single-group study with continuous variables represented as mean, standard error, standard deviation, and confidence interval, which are shown in the analysis of AUC and BA plots. The number of 5 is reasonable, as shown in previous studies. Also, this was a comparison of saturations at different locations with multiple time points corresponding to the arterial blood gas analysis using a single group. An NM3 Philips monitor was used to monitor tidal volume, and peak inspiratory pressures were adjusted to target a tidal volume of 7 to 9 mL/kg with respiratory rates of 40 per minute. We used initial supplemental oxygen of 60% and titrated it after 2 min based on Neonatal Resuscitation Program (NRP)-recommended preductal SpO_2_ as mentioned previously per our protocol [13]. The 2 min delay was to ensure that the preductal SpO_2_ was detected. The study evaluated HR and SpO_2_ using preductal, postductal, and umbilical cord pulse oximetry probes for 10 min. Although the cord was cut and a clamp was placed to prevent bleeding, the pulse ox probe was left on the umbilical cord towards the fetal side, and SpO_2_ and HR were recorded for 10 min. We also present a pulsality of UA and UV post-cord clamp at 4 min.

### 2.4. Data Analysis

XLSTAT 2024 (Lumivero, CO, USA) was used to analyze the receiver operator characteristic curve and the Bland–Altman (BA) plot. Receiver operator characteristic (ROC) curve—Arterial blood gas analysis served as the gold standard for oxygen saturation (SaO_2_), and carotid artery measurements were used as the reference for HR to analyze umbilical SpO_2_ and HR. Values after 2 min until 10 min were used for the ROC. The area under the curve above 0.5 with a probability value of <5% was considered significant. The BA plot shows the agreement and bias between the two variables.

## 3. Results

### 3.1. Feasibility

Table 1 shows the characteristics of the five lambs, which were similar. Resuscitation was initiated with 60% oxygen and titrated based on NRP guidelines. The supplemental oxygen used in the first 10 min during PPV was 60 (CI 21–100) %.

**Figure 1:**
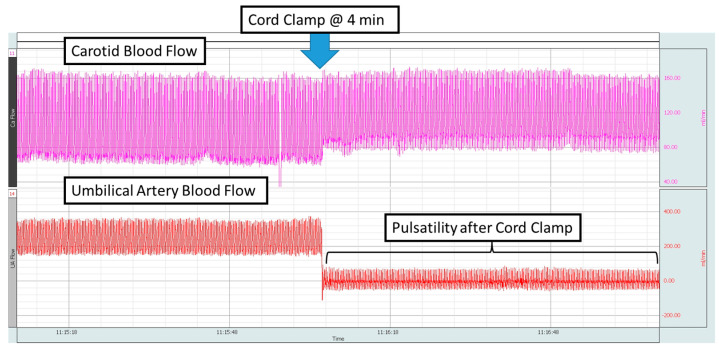
The BIOPAC snapshot shows the carotid artery flow and the umbilical arterial flow with pulsations during the 4 min delayed clamping of the cord and post-clamping. The downward blue arrow shows the time point when the cord was clamped, and the absence of flow in the umbilical artery can be observed with pulsatility. Purple—carotid blood flow, red—umbilical artery flow.

A BIOPAC snapshot (Figure 1) that shows the pulsatile flow in the carotid artery and from the flow transducer placed around the umbilical artery (UA). Figure 1 shows the drop in blood flow in the UA after the cord is clamped, with the remaining pulsatile activity that allows for U SpO_2_ and heart rate measurements to be possible after the cord is cut.

**Figure 2:**
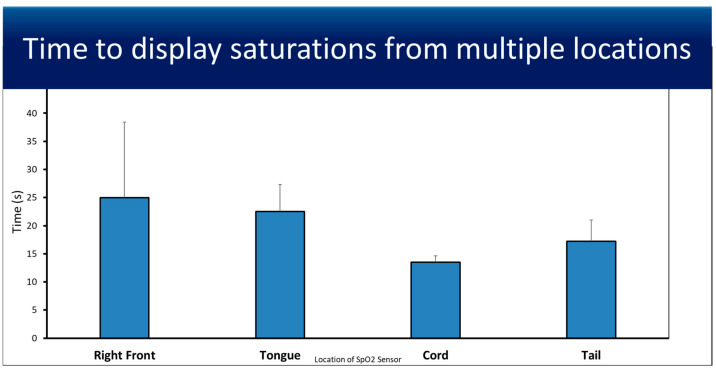
The bar chart shows the time from placement of the sensor to first display of SpO_2_ on the Nonin X-100 Sensmart monitor from four measurement locations: right front limb, tongue, umbilical cord, and tail. Times are represented as averages and standard deviations from 4 placements at each measurement location. The umbilical cord had the shortest time (13.5 s ± 1.12) compared to the right front limb (25 s ± 13.44), tongue (22.5 s ± 4.82), and tail (17.25 s ± 3.77), but the differences were not significantly different.

The average time from SpO_2_ sensor placement until saturation and the heart rate were displayed by the Nonin X-100 Sensmart from all four measurement locations (Figure 2). Each location was tested four times, with U SpO_2_ having the fastest average time to display saturation (13.5 s ± 1.12), although not significantly different.

**Figure 3:**
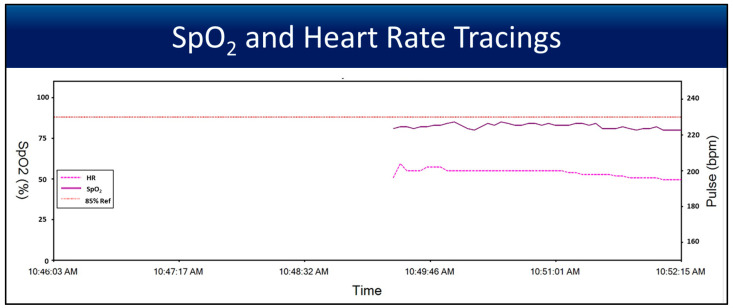
The line graph shows that when the Nonin sensor is placed on the cord, it quickly achieves a stable measurement for both heart rate and SpO_2_. Both measurements correlate with preductal and postductal sensors.

Figure 3 shows a tracing of SpO_2_ and heart rate measurements from a Nonin sensor placed on the umbilical cord. The tracings show how quickly a stable and reliable measurement for both U SpO_2_ and U HR can be achieved when an SpO_2_ sensor is placed on the umbilical cord.

### 3.2. Saturations

**Figure 4:**
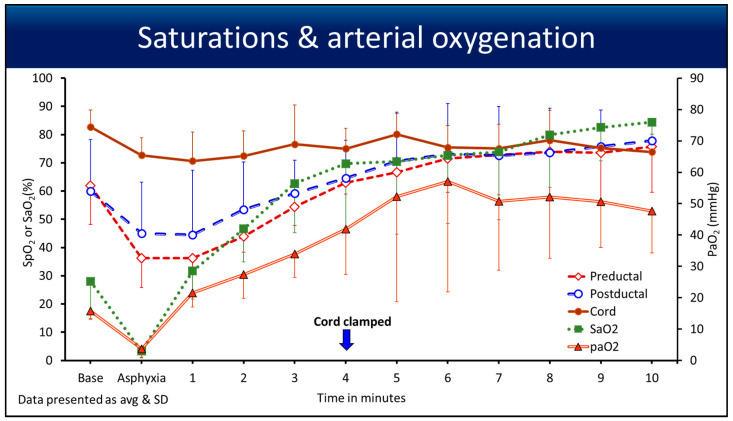
The line graph shows the preductal (diamond shape), postductal (open circle), cord (closed circle), and SaO_2_ (arterial saturation) (square) as percentage values on the *y*-axis with events on the *x*-axis. The PaO_2_ (arterial oxygenation) (triangle) in mmHg is shown on the secondary *y*-axis. The data are represented as averages and standard deviations. The standard deviations are represented in one direction to avoid confusion. The downward arrow shows when the delayed cord clamping ended, and the cord was clamped, but the umbilical pulse ox probe was left in place for 10 min.

Oxygen saturation curves from baseline until 10 min, along with arterial oxygenation (PaO_2_) on the secondary *y*-axis, are shown in Figure 4. The U SpO_2_ remained stable post-asphyxia, picking up the flow from the umbilical cord. The SaO_2_ (%) and the PaO_2_ in mmHg had a drop during asphyxia with an HR < 90 bpm (from a carotid artery) compared to pre, post, and umbilical SpO_2_.

In the first 2 min after PPV was initiated with an intact cord, the failure rate at which measurements could not be made was 18% in the pre- and postductal regions and 8% in the U SpO_2_ region. This is consistent with the manufacturer’s failure rate in challenging situations, such as low perfusion in a bradycardic surfactant-deficient preterm ovine model. After 2 min with an increase in perfusion, the SpO_2_ picked up, as shown in Figure 4.

**Figure 5:**
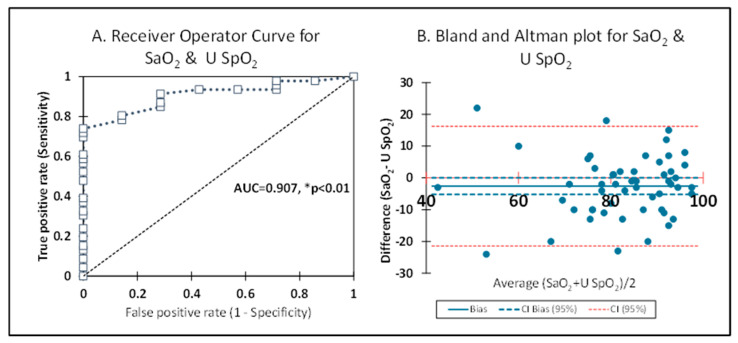
(**A**) The receiver operator curve was constructed by plotting the true positive rate against the false positive rate for the umbilical cord (U SpO_2_) and arterial saturation (SaO_2_) as the gold standard. The area under the curve was highly significant (AUC = 0.907, * *p* < 0.0001). (**B**) The Bland and Altman plot shows the averages of SaO_2_ and U SpO_2_ on the *x*-axis and the differences between them on the *y*-axis.

ROC curve—Figure 5A. With SaO_2_ as the gold standard from arterial blood gas, U SpO_2_ after 2 min had an area under the curve (AUC) of 0.907 (CI 0.857–0.968), with an accuracy of 0.923, *p* < 0.0001, which was significant (Figure 5A). Preductal SaO_2_ and U SpO_2_ had an AUC of 0.543 (CI 0.365–0.691), with an accuracy of 0.556, which represents poor signal pick-up in the preductal SpO_2_ probe. BA plot—Figure 5B shows the bias between U SpO_2_ and SaO_2_, which was −5.2 (95% CI −10 to −0.5). This −5.2 is closer to zero, with >95% of the data falling between 2 standard deviations (95% CI), which is considered to be in agreement with the gold standard (SaO_2_) and experimental intervention (U SpO_2_). These findings using the ROC and BA plot suggest that U SpO_2_ measurement could be helpful clinically for oxygen saturation assessment, especially to guide supplemental oxygen exposure.

### 3.3. Heart Rate

**Figure 6:**
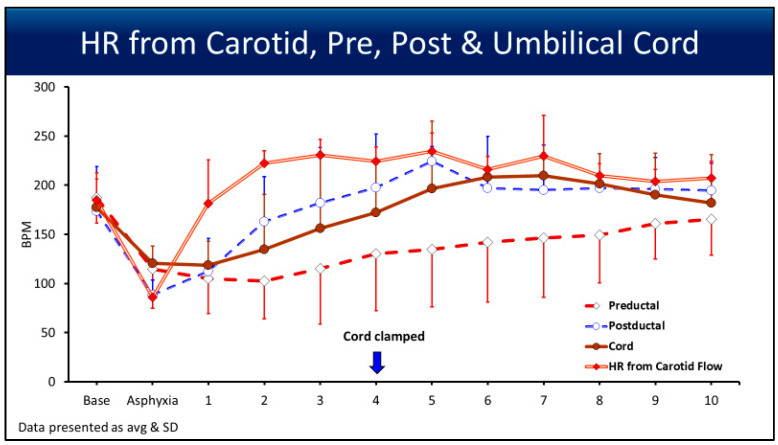
The line graph shows the heart rate (HR) obtained from the preductal (diamond), postductal (open circle), cord (closed circle), and carotid arteries (closed diamond), represented as BPM on the *y*-axis, with the *x*-axis showing the events. The data are represented as averages and standard deviations. The standard deviations are represented in one direction to avoid confusion. The downward arrow shows when the delayed cord clamping ended and the cord was clamped, but the umbilical pulse ox probe was left in place for 10 min.

Heart rate (BPM) changes from baseline until 10 min were recorded (Figure 6). The carotid HR at asphyxia was 85 ± 7 bpm, as shown. The pulse oximetry readings of the HR during asphyxia were not accurate. Two minutes after asphyxia and PPV, HR detection improved with all four pulse oximetry readings.

**Figure 7:**
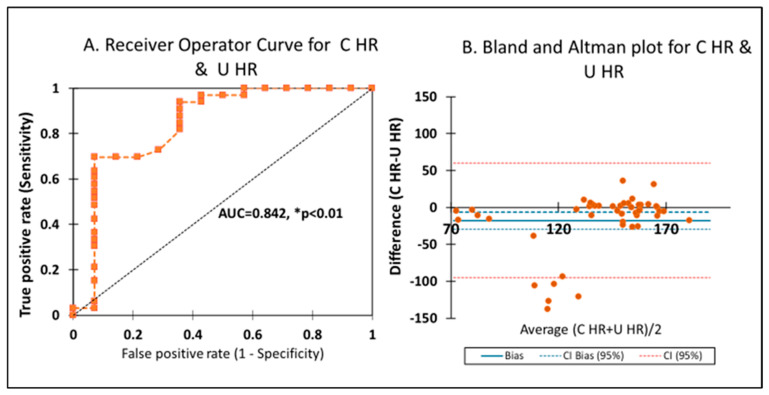
(**A**) The receiver operator curve was constructed by plotting the true positive rate against the false positive rate for umbilical cord heart rate (U HR) and carotid heart rate (C HR) as the gold standard. The area under the curve was significant (AUC = 0.842, * *p* < 0.0001). (**B**) The Bland and Altman plot shows the averages of U HR and C HR on the *x*-axis and the differences between them on the *y*-axis.

ROC curve—Figure 7A—With carotid HR as the gold standard, U HR after 2 min had an area under the curve of 0.842 (CI 0.663–0.902), *p* < 0.0001, with an accuracy of 0.701, which was significant (Figure 7A), although carotid HR was higher compared to U HR. BA plot—Figure 7B shows the bias between U HR and carotid HR, which was −9.4 (95% CI −20 to 1.5). The −9.4 is closer to zero, with >95% of the data points falling between 2 SDs (CI 95%), which is significant for the BA plot. Since invasive HR monitoring is not always available in the delivery room, an HR with a bias of <10 bpm and a tight confidence interval shows agreement between a gold standard and experimental method. These findings using the ROC and BA plot suggest that U HR measurement could be helpful clinically for HR assessment, especially to differentiate pulseless electrical activity when ultrasound is not available.

## 4. Discussion

A pulse oximeter reading is a transcutaneous, non-invasive estimate of oxygen saturation based on the principle that oxyhemoglobin and deoxyhemoglobin differentially absorb red and near-infrared light [14]. This is the first study to explore the concept of umbilical cord pulse oximetry, which is novel. The study highlights the potential for umbilical cord pulse oximetry to enhance neonatal resuscitation practices. Post-COVID, Sjording et al. noted that skin color interferes with saturation readings [8]. They found that black patients had nearly three times the frequency of occult hypoxemia that was not detected by pulse oximetry compared to white patients. In February 2021, the FDA announced the factors that could affect pulse oximetry readings [6]. Now considered the ‘fifth vital sign,’ oxygen saturation in neonates can be affected in the delivery room by skin color, low temperature, and low perfusion. Preterm neonates are at risk of all of the above, and with surfactant deficiency, supplemental oxygen continues to be a subject of debate [1,15]. Although postductal, bypassing skin-related factors that may affect pre- and postductal oximetry could provide quicker and more reliable data in the critical minutes after birth. When there is interference from external light sources, such as the radiant warmer, especially in a dark-skinned newborn, it could disrupt signal processing with preductal SpO_2_, in which case U SpO_2_ could be useful.

The high agreement between umbilical cord SpO_2_ and SaO_2_ with less bias supports its use for assessing oxygen saturation in the delivery room. U SpO_2_ could help optimize oxygen supplementation and avoid skin and other factors in a preterm infant in the delivery room, as a saturation of <80% and an HR < 100 bpm have shown higher morbidity and mortality [16,17]. Acrocyanosis is a common finding in air-breathing term neonates, which reduces the Apgar score to 9 most often [18]. Low perfusion to the peripheries (arms and legs) can occur more frequently in preterm infants, especially in the setting of temperature dysregulation, with the need for polyvinyl bags and other interventions to prevent heat loss and conserve body temperature. Proper placement of the pulse oximeter probe is often challenging, and when positioned correctly, it typically takes 1 to 2 min for preductal SpO_2_ readings to stabilize [1,15]. With the luxury of the umbilical cord in newborns, pulse oximetry in the cord could be valuable, especially in the first few minutes of life. In our study, we noticed that U SpO_2_ rapidly displayed measurements (Figure 2) and remained steady immediately after asphyxia as the umbilical flow was established (Figure 3). Initially, oxygenated blood from the ‘ewe/maternal lamb’ could have influenced these readings. This is another reason that the values for analysis were taken after 2 min to reflect the lamb’s SpO_2_.

The highest average arterial oxygenation was 57 mmHg in this study, which could be influenced by deferred cord clamping and the lack of surfactant administration until after 11 min of PPV. Previous studies have noted that the relationship between SpO_2_ and PaO_2_ is asymptotic [14,19]. For a given PaO_2_, the SpO_2_ values differ, and this difference is even higher for high arterial oxygenation. We did appreciate the differences between pre, post, and umbilical SpO_2_ compared to PaO_2_ and have shown the trend in Figure 4. Hence, SpO_2_, regardless of its placement site, may not accurately predict the oxygen load on tissues.

### 4.1. Point of Interest: How Can You Measure the SpO_2_ and HR for 10 min if the Cord Is Clamped at 4 min?

We were able to measure the SpO_2_ and HR for 10 min even when the umbilical cord was cut and clamped at 4 min. The BIOPAC snapshot (Figure 1) shows the pulsatility of carotid and umbilical artery flow after the cord was clamped at 4 min. The blood flow from the fetus to the placenta stops after the cord is clamped and cut. The clamp was placed to avoid bleeding from the pulsatile arterial vessels. Since pulse oximetry measures the oxygenated hemoglobin and displays the saturations, and pulsatile flow helps to determine the HR, umbilical pulse oximetry with the probe placed towards the fetus can accurately predict these two parameters for over 10 min. Moreover, the umbilical artery can be catheterized from the first few days of life to almost a week, which shows its patency.

We used carotid HR to assess umbilical HR accuracy with a significant area under the curve. Heart rate is best measured by electrocardiogram (EKG) [2,3,20]. We did not place an EKG in this model, since we had an invasive carotid arterial line to measure HR accurately. For preterm neonates whose heart rate responds to PPV with a stable HR, umbilical pulse oximetry that measures U HR (umbilical HR) could avoid further interventions in the delivery room and improve bonding with the parent in the delivery room. Since HR is detected, as stated previously, by sensing pulsatile blood flow, umbilical pulse oximetry could be used to confirm umbilical blood flow. The agreement with the ROC curve and the low bias seen on the BA plot supports its use to detect HR in the delivery room.

### 4.2. Point of Interest: Can Umbilical Cord Pulse Oximetry Be Helpful to Determine the Duration of Deferred Cord Clamping?

A systematic review and meta-analysis have shown that a longer deferral of umbilical cord clamping ≥ 120 s reduced the odds of death in preterm infants before discharge [21]. With evidence-based management taking priority, subjective assessment of vital signs, such as HR and color/perfusion has been objectively replaced by EKG and pulse oximetry, guiding us through neonatal resuscitation’s next steps. Umbilical pulse oximetry could be the objective parameter, which may help us assess the umbilical flows by assessing the HR and verifying the pulsatility of the umbilical artery (Figure 1). We are conducting studies using the ovine model to show the parameters to differentiate umbilical pulse with and without blood flow. The umbilical venous and umbilical arterial blood flows demonstrated for the first 4 min were not different, and the net flow was zero, as shown in our previous studies [13,22]. Nevertheless, Figure 1 shows the drop in blood flow and the presence of continued pulsatility after the cord is clamped, which could be predicted with flow indices. Deferred cord clamping could be performed with umbilical pulse oximetry, as long as stable HR and saturations are recorded in conjunction with the clinical picture of an asphyxiated newborn. In a scenario with possible asphyxia, especially due to an umbilical cord issue, a saturation probe secured on the umbilical cord close to the newborn’s abdomen, regardless of deferred or immediate clamping, will give us a reliable signal, as shown in Figure 1, if the cardiovascular function is intact with HR and flows, causing pulsatility. We expect the initial U SpO_2_ and U HR to be similar, as shown in Figure 4 and Figure 6, in relation to the cord hemodynamics.

We acknowledge the limitations of our study, one of which is the inherent species differences, and we emphasize the differences, such as a short umbilical cord with two arteries and two veins. However, this model is well established and has been used in studies evaluating cord management, such as deferred cord clamping (the net flow to the fetus could be zero) and cord milking. Our study was done in a controlled manner, and asphyxia was induced along with 4 min deferred cord clamping. The ability to record SpO_2_ and HR for up to 10 min after cord clamping at 4 min and its clinical relevance—particularly in the absence of neonatal hemodynamics or when only residual pulsatility remains in the clamped cord—need validation in newborn infants. We chose the gold standard for both SpO_2_ and HR, as invasive measurements are more accurate. But as the reviewer has pointed out, the absence of EKG is a methodological limitation, as it is the preferred method of assessing HR in the delivery room. This model of asphyxiated preterm lamb is delivered by cesarean section under general anesthesia without uterine contractions, and hence, there is no net umbilical flow. These observations could differ in neonates born via normal delivery and breathing air with uterine contractions. Deferred or delayed cord clamping can increase the SpO_2_ and a higher nomogram, as shown in normal air-breathing newborns compared to infants with immediate cord clamping [23,24,25]. In the context of DCC and blood transfusion from the placenta to the fetus, previous studies have shown that PPV, as well as c-section without uterine contraction, may limit the blood volume transferred. This study shows that even with that potential limitation on the blood volume in the cord, U SPO_2_ was still able to provide accurate and reliable measures. Further studies with human neonates are required to confirm these findings in births with spontaneous breathing and uterine contractions. We acknowledge the small number of five preterm lambs. Based on a study using a Markov Chain Monte Carlo (MCMC) approach to estimate the sample size for pilot animal experiments, a common practice of using five or six animals per group for continuous endpoints is reasonable. [11] Even in the case of small effect sizes, the statistical power would be sufficiently large (≥80%). The MCMC approach has been demonstrated to be a useful method for calculating sample size in animal studies that lack prior data. Regardless, the type II error in a controlled single-arm study is an estimate and needs validation. But this is a single-arm study, and we did have minute-to-minute data points, and larger studies are needed and are ongoing. [11] No clinical recommendations should be changed based solely on a translational model. Our ovine model is well established in studies looking at the hemodynamics of transition, specifically pulmonary, as well as cord management, due to the similar size and fetal development of the lungs and vasculature. Clinical studies in human neonates are required to confirm our results as well as feasibility. We speculate that the umbilical cord SpO_2_ could be higher, similar to previous clinical studies, when used during deferred cord clamping.

Future direction:

We are conducting further studies using ovine models to establish parameters for deferred cord clamping. Pilot studies are being conducted in normal air-breathing newborns with umbilical pulse oximetry to assess feasibility. With the correct technique and proper implementation strategies, placing a pulse ox probe in the delivery room could be feasible, and it can be used as an objective measure of umbilical flows.

## 5. Conclusions

In a translational preterm model, umbilical cord pulse oximetry accurately predicted pre-ductal SaO_2_ and carotid HR (a surrogate for umbilical flows). Using UCP in the delivery room may avoid excess oxygen exposure and help confirm pulsatile flow in the umbilical cord, which could help determine the duration of deferred cord clamping. This innovative technique presents a promising alternative for monitoring oxygen saturation and heart rate in neonates, particularly preterm infants, in the delivery room. This method could complement current preductal and postductal oximetry techniques by offering faster and more reliable data without interference from skin factors. As mentioned in the future directions, the initial feasibility/pilot study should focus on securing the saturation probe around the umbilical cord and assessing the agreement between the preductal SpO_2_/HR and U SpO_2_/HR, which could be a technical but not an ethical challenge, as the umbilical cord is not considered sterile unless cleaned and cut to place umbilical catheters. Further, clinical studies paying attention to securing the pulse oximetry probe on the cord, without interfering with the resuscitation of newborns, are needed to validate this proof-of-concept study and explore the practical applications of umbilical cord pulse oximetry in human neonates.

## Figures and Tables

**Table 1:** Characteristics of the five lambs used in this study were similar.

Multiplicity	Weight (kg)	Gestational Age (days)	Gender
Twin	2.75	126	M
Single	3.1	125	M
Single	3.31	126	F
Single	3.8	126	F
Single	2.47	126	M

## Data Availability

Ongoing study. Data will be available upon reasonable request once the study is completed.

## Abbreviations

The following abbreviations are used in this manuscript:

UCP	umbilical cord pulse oximetry
SpO_2_	preductal saturations
SaO_2_	arterial saturations
U SpO_2_	umbilical saturations
C HR	carotid heart rate
U HR	umbilical heart rate
CA	carotid artery
UV	umbilical vein
UA	umbilical artery
